# Adverse drug reactions in an ageing PopulaTion (ADAPT) study: Prevalence and risk factors associated with adverse drug reaction-related hospital admissions in older patients

**DOI:** 10.3389/fphar.2022.1029067

**Published:** 2023-01-13

**Authors:** Caitriona Cahir, Carmel Curran, Caroline Walsh, Anne Hickey, Ross Brannigan, Ciara Kirke, David J. Williams, Kathleen Bennett

**Affiliations:** ^1^ Data Science Centre, School of Population Health, RCSI University of Medicine and Health Sciences, Dublin, Ireland; ^2^ Department of Geriatric and Stroke Medicine Beaumont Hospital, Dublin, Ireland; ^3^ National Centre for Pharmacoeconomics, St. James’s Hospital, Dublin, Ireland; ^4^ Discipline of Pharmacology and Therapeutics, School of Medicine, Trinity College Dublin, The University of Dublin, Dublin, Ireland; ^5^ Department of Psychology, School of Population Health, RCSI University of Medicine and Health Sciences, Dublin, Ireland; ^6^ National Quality and Patient Safety Directorate at Health Service Executive, Dublin, Ireland; ^7^ Department of Geriatric and Stroke Medicine, RCSI University of Medicine and Health Sciences, Dublin, Ireland

**Keywords:** adverse drug reaction, older people, hospital admission, medication, risk factors

## Abstract

**Background:** Older people experience greater morbidity with a corresponding increase in medication use resulting in a potentially higher risk of adverse drug reactions (ADRs).

**Objectives:** The aim of this study was to; 1) determine the prevalence and characteristics of ADR-related hospital admissions among older patients (≥65 years) in Ireland; and 2) identify the risk factors associated with ADR-related hospital admissions.

**Methods:** A cross-sectional study of ADR prevalence in patients aged ≥65 years admitted acutely to hospital in Ireland over a 8 month period (November 2016- June 2017). A multifaceted review of each hospital admission was undertaken to assess the likelihood of an ADR being a reason for admission (cause of admission or contributing to admission) in the context of the patient’s medication, clinical conditions, comorbidities and investigations. A number of decision aids were applied by two independent reviewers to assess ADR causality, avoidability and severity. A random sample of patients, determined not to have a suspected ADR on screening, were assigned to a non-ADR control group. Multivariable logistic regression was used to assess the association between potential risk factors for ADR-related admissions compared with non-ADR-related admissions.

**Results:** In total, 3,760 hospital admission episodes (in 3,091 patients) were screened and 377 admissions were considered ADR-related (10.0%, 95% CI 9.1%, 11.0%). 219 (58.1%) ADR-related admissions were caused by an ADR, while ADRs contributed to 158 (41.9%) admissions. 268 (71.1%) of all ADR-related admissions were deemed definitely or possibly preventable/avoidable. 350 (92.8%) ADRs were classified as being of moderate severity, with 27 (7.2%) classified as severe. Antithrombotic agents, mainly aspirin and warfarin, were the drugs most frequently associated with ADR-related admissions (gastrointestinal and vascular haemorrhagic disorders). In multivariable analysis, immobility, frailty, having delirium or ulcer disease and taking anticoagulant and antiplatelet medication on admission were significantly associated with an ADR-related hospital admission.

**Conclusion:** One in ten hospital admissions, among those aged 65 + years, were considered ADR-related, with approximately 70% potentially avoidable. Reliable and validated ADR detection and prediction tools are needed to develop prevention strategies.

## Introduction

An adverse drug reaction (ADR) is defined as ‘an appreciably harmful or unpleasant reaction resulting from an intervention relating to the use of a medicinal product’ (Edwards and Aronson, 2000). Older people experience greater morbidity, increased medication utilisation and a variety of physiological changes affecting the pharmacokinetics and pharmacodynamics of medications and are therefore at an increased risk of ADR-related hospital admissions (Franceschi et al., 2008; Lehnert et al., 2011). Two systematic reviews have suggested a median ADR-related hospital admission rate of 10% and 11%, respectively, in those aged ≥65 years (Kongkaew et al., 2008; Alhawassi et al., 2014). A meta-analysis of observational studies measuring hospitalisations due to ADRs found one in ten hospital admissions of older patients to be due to an ADR (Oscanoa et al., 2017). However both reviews and the meta-analysis have reported wide variation in ADR prevalence rates, ranging from 5% to 50%, with heterogeneity in how ADRs are defined and identified given as the principle reason for much of this variability (Alhawassi et al., 2014).

ADRs are difficult to identify in older populations and hospital reporting systems significantly under-report the incidence of ADRs resulting in unreliable estimates of ADR-related hospital admissions in older populations (Waller et al., 2004; Sari et al., 2007). A prospective review classified 15% of medical admissions to be ADR-related in older people, compared with 2.7% in the same patient cohort using administrative coding (Parameswaran Nair et al., 2017). To accurately detect ADRs, a number of methods are required including an in-depth medical record review and a causality assessment between the drug and the adverse clinical event (Williams et al., 2008).

While studies have been performed in the United Kingdom, Europe and the United States, there is limited data published on the prevalence and characteristics of ADR-related hospital admissions in older people in Ireland (Kongkaew et al., 2008; Alhawassi et al., 2014; Osanlou et al., 2022). A 4-week study of ADR-related hospital admissions in the general adult population reported a prevalence rate of 8.8%, with over half deemed preventable (Ahern et al., 2014). Internationally studies have indicated that more than half of ADR-related hospital admissions in older patients are preventable with only 19%–28% of ADRs considered unavoidable (Pirmohamed et al., 2004; Franceschi et al., 2008). Identifying the characteristics of ADR-related hospital admissions, including the types of drugs involved and the nature of the harm represents an important gap in knowledge in preventing ADR-related hospital admissions.

Another approach to preventing ADR-related hospital admissions in older patients is to identify those who are most at risk of ADR-related admissions. Previous risk prediction tools have mainly focused on ADRs occurring within the hospital setting and few have been developed for use in community settings (Onder et al., 2010). A systematic review identified age, female gender, increasing comorbid burden and number of medications to be associated with an increased ADR risk in older people in the acute care setting (Alhawassi et al., 2014). However, the list of risk factors investigated was not comprehensive and other factors, such as functional and social factors, may contribute to ADR-related hospitalisation. The aims of our study were to; 1) determine the prevalence and characteristics of ADR-related hospital admissions among older patients (≥65 years) in Ireland; and 2) identify the risk factors associated with ADR-related hospital admissions.

## Methods

### Study design

This was a cross-sectional study of ADR prevalence in all patients aged ≥65 years admitted acutely to a large tertiary referral hospital in Ireland over a 8 month period (November 2016-June 2017). The study protocol has previously been published (Cahir et al., 2017). Ethical approval was obtained from Beaumont Hospital Ethics Committee (REC 16/49).

### ADR screening

All admitted patients were screened for a suspected ADR-related hospital admission within the first 36 h of admission by the research team (Consultant Geriatrician (CCu), two hospital pharmacists (CW, CB)) and a research nurse (ML)) using a previously validated screening process (Pirmohamed et al., 2004; Hopf et al., 2008). Patients were excluded if they were transferred from other hospitals, were elective non-acute admissions or aged under 65 years. The screening approach incorporated a multifaceted review of each hospital admission to assess the likelihood of the ADR being a reason for admission (cause of admission or contributing to admission) in the context of the patient’s medication, clinical conditions, medical history, comorbidities and investigations. A number of independent sources were consulted to verify the patient’s medication history, including the patient’s self-reported medication list, pharmacist medication list and general practitioner (GP) medication list. The medication list included recently discontinued or short-course medications, over-the-counter (OTC) medications and herbal preparations as part of the medication reconciliation process. A random sample of patients, who were determined not to have a suspected ADR on screening, were assigned to a non-ADR control group for comparative purposes. These patients were randomised to the non-ADR control group from the hospital admission list, which detailed patients’ chronological order of hospital admission on each day for those aged ≥65 years, using randomisation software http://www.randomization.com.

### ADR characteristics

Two members of the research team (CCu and CW or CB) independently applied a number of decision aids and validated algorithms to assess the causality, preventability and severity of each ADR. ADR causality was determined using the World Health Organisation (WHO) criteria, the Naranjo criteria and the Liverpool Algorithm (Naranjo et al., 1981; WHO, 2005; Gallagher et al., 2011). The Hallas criteria were used to categorise the avoidability/preventability of the ADRs (Hallas et al., 1990). ADR severity was classified using the Hartwig severity assessment scale (Hartwig et al., 1992). Differences in causality, preventability and severity between the two reviewers were reviewed by an independent third reviewer (DW; Clinical Pharmacologist). The nature of the reaction was reported using the Medical Dictionary for Regulatory Activities (MedDRA) terminology (WHO, 2005). The details of all medications involved in the ADR-related hospital admission were recorded using the WHO Anatomical Therapeutic Chemical (ATC) codes.

### Risk factors associated with ADR-related hospital admissions

A number of potential risk factors for an ADR-related hospital admission were measured as part of the ADR screening process on hospital admission. They were categorised as (Edwards and Aronson, 2000); sociodemographic-related risk factors (Franceschi et al., 2008); functional ability-related risk factors (Geriatric syndromes) (Lehnert et al., 2011); disease-related risk factors and (Kongkaew et al., 2008); medication-related risk factors. Sociodemographic risk factors included age, gender and medical card status (Yes/No). Medical card eligibility is means-tested and entitles the individual to free or substantially-subsidised healthcare (Sinnott et al., 2017). Functional ability-related risk factors (Geriatric syndromes) included measures of mobility, functional impairment, falls, frailty, delirium, urinary incontinence (Yes/No), unintentional weight loss in the previous 6 months (Yes/No) and nursing home residency (Yes/No) (Cahir et al., 2017). Patients self-reported if they were immobile (Yes/No), their level of mobility (use of walking aids when crossing a room and when outside), if they had a functional impairment and their falls history (fallen previously, fallen in the last year, fallen more than once). Frailty was assessed using the Triage Risk Screening Tool (Fan et al., 2006) and the PRISMA-7 (Hebert et al., 2010). Delirium was assessed using the 4AT (De et al., 2016) and DSM4 criteria (American Psychiatric Association, 1994). Disease-related risk factors included certain diagnoses (e.g. chronic lung disease, cerebrovascular disease) and comorbidity burden was measured using the Charlson co-morbidity index (Charlson et al., 1987). Medication-related risk factors included number and types of medications, polypharmacy, use of blister packs (Yes/No) and self-reported adherence (Yes/No). Polypharmacy was defined as greater than five medications and excessive polypharmacy as greater than 10 (Dwyer et al., 2016).

### Data analysis

#### Prevalence and characteristics of ADR-related hospital admissions

Descriptive statistics, including median (inter-quartile range (IQR), percentages and frequencies, as appropriate, with 95% confidence intervals (CIs), were used to summarise the results of the prevalence of ADRs, their various classifications (e.g., preventability, severity) and the drug classes involved in ADRs. Cohen’s Kappa statistics (κ) were used to measure inter-rater reliability between the two reviewers, on the measures of causality, preventability and severity of each ADR, with interpretation as follows; poor (<0.20), fair (0.20–0.40), moderate (0.41–0.60), good (0.61–0.80), and very good (0.81–1.00). (Altman, 1991). The primary presenting complaint in ADR-related and non-ADR related hospital admissions were compared using chi-square tests for categorical variables, with Bonferroni corrections (*p* < 0.003).

#### Risk factors associated with ADR-related hospital admissions

Descriptive statistics including means (standard deviation, SD), medians (IQR) and proportions, were calculated for all risk factors. The associations between all risk factors and ADR-related hospital admissions *versus* non-ADR-related admissions were assessed using a multivariable logistic regression model. Adjusted ORs with 95% CIs are presented. The data was analysed using SAS Version 9.4 statistical package and Stata Version 17.0 (StataCorp, College Station, TX, United States). Adjusted ORs with 95% CIs are presented. Significance at *p* < 0.05 is assumed. The data was analysed using SAS Version 9.4 statistical package and Stata Version 17.0 (StataCorp, College Station, TX, United States). Significance at *p* < 0.05 is assumed.

## Results

### Prevalence and characteristics of ADR-related hospital admissions

A total of 3,760 hospital admission episodes (in 3,091 eligible patients), were screened for an ADR and 377 were determined to be ADR-related (10.0%, 95% CI 9.1%, 11.0%); 41 (10.9%) of these ADR-related admissions were related to ≥2 ADRs (*n* = 424 total ADRs). Of the 377 ADR-related admissions, 219 (58.1%) admissions were caused by an ADR, while ADRs contributed to 158 (41.9%) admissions. For the majority of the ADRs (N = 229, 54.0%) there was no other known acute medical issue that may have contributed to the ADR.

There was moderate agreement between the two reviewers as per the WHO criteria (*κ* = 0.54) and good agreement as per the Naranjo criteria (κ = 0.65) and Liverpool Algorithm (*κ* = 0.71). There was also good agreement regarding preventability as per the Hallas criteria (κ = 0.73) and very good agreement for severity as per the Hartwig severity assessment scale (*κ* = 0.98). Table 1 presents the overall causality of the ADRs according to the three sets of criteria. The majority of ADRs were deemed possible ADRs (66%–77%), with approximately one-fifth classified as probable/likely ADRs. Forty-three (11.4%) ADRs were deemed definitely preventable/avoidable, 225 (59.7%) possibly preventable/avoidable and 109 (28.9%) unavoidable. In total, 350 (92.8%) ADRs were classified as being of moderate severity, with 27 (7.2%) classified as severe.

**TABLE 1:** Classification of ADR causality per the WHO criteria, Naranjo criteria and Liverpool Algorithm (*n* = 424).

	Possible ADR	Probable/likely ADR	Certain/Definite ADR	Unlikely/doubtful ADR
	N (%)	N (%)	N (%)	N (%)
WHO criteria	309 (72.9)	87 (20.5)	26 (6.1)	2 (0.5)
Naranjo criteria	328 (77.4)	87 (20.5)	9 (2.1)	0 (0)
Liverpool Algorithm	281 (66.3)	99 (23.4)	44 (10.4)	0 (0)

Table 2 identifies the most frequent classes of drugs associated with the ADR-related hospital admissions and the nature of the reaction. Antithrombotic agents, mainly aspirin and warfarin, were the drugs most frequently associated with ADR-related hospital admissions with 33% of ADR-related hospital admissions citing gastrointestinal haemorrhage and vascular haemorrhagic disorders as the main adverse reactions. A number of cardiovascular system drugs were associated with ADR-related hospital admissions including diuretics, agents acting on the renin-angiotensin-aldosterone system, calcium channel blockers and beta-blocking agents (ranging from 32% to 6% of ADR-related admissions respectively). These drugs were associated with the adverse reactions of hypotension and non-specific blood pressure disorders, and shock and electrolyte and fluid balance conditions. Psychoanaleptics (6% of ADR-related hospital admissions) were associated with the adverse reactions of electrolyte and fluid balance conditions. Supplementary Table 1 provides further detail on the diagnostic categories associated with ADR-related hospital admissions.

**TABLE 2:** The main classes of drugs associated with ADR-related hospital admissions and the nature of the reaction (*n* = 424).

Therapeutic group (ATC)	N (%)	Main drug substances	N (%) of therapeutic group^a^	Nature of the ADR^b^	N (%) of therapeutic group^a^
Antithrombotic agents (B01)	141 (33)	Aspirin	77 (55)	Gastrointestinal –haemorrhage and inflammatory conditions	70 (50)
		Warfarin	28 (20)	Vascular- haemorrhagic disorders	45 (32)
		Aspirin and Warfarin	19 (13)		
Diuretics (C03)	134 (32)	Furosemide	65 (49)	Renal disorders	62 (46)
		Hydrochlorothiazide	21 (16)	Electrolyte and fluid balance conditions	48 (36)
		Bumetanide	21 (16)	Hypotension and non-specific blood pressure disorders and shock	23 (17)
Agents acting on the renin-angiotensin-aldosterone system (C09)	127 (30)	Ramipril	37 (29)	Renal disorders	56 (44)
		Perindopril	27 (21)	Hypotension and non-specific blood pressure disorders and shock	33 (26)
		Valsartan	18 (14)	Electrolyte and fluid balance conditions	32 (25)
Calcium channel blockers (C08)	28 (7)	Amlodipine	17 (61)	Hypotension and non-specific blood pressure disorders and shock	18 (64)
Beta-blocking agents (C07)	26 (6)	Bisoprolol	18 (69)	Hypotension and non-specific blood pressure disorders and shock	12 (46)
				Cardiac arrhythmia	11 (42)
Psychoanaleptics (N06)	25 (6)	Escitalopram	6 (24)	Electrolyte and fluid balance conditions	13 (52)

^a^ Number and percentage of ADRs, associated with a drug substance as a proportion of the overall number of ADRs, associated with the therapeutic drug group during the study period.

^b^ The nature of the reaction is reported using Medical Dictionary for Regulatory Activities (MedDRA) terminology and refers to the therapeutic group.

Table 3 compares the primary presenting complaint in ADR-related and non-ADR related hospital admissions. In ADR-related hospital admissions, there was a significantly higher proportion of bleeding disorders, gastrointestinal disorders and syncope and hypotension compared to non-ADR hospital admissions. There was a significantly higher proportion of respiratory, cardiac and muscoskeletal disorders in the non-ADR group (*p* < 0.003).

**TABLE 3:** Primary presenting complaint at hospital admission (*n* = 814).

Primary presenting complaint at hospitalisation	Total (*n* = 814)	Non-ADR admissions (*n* = 437)	≥1 ADR admissions (*n* = 377)	*p*-value^a^
	N (%)	N (%)	N (%)	
*Respiratory disorders*	173 (21.3)	110 (25.2)	63 (16.7)	*p* < 0.003^a^
*Bleeding disorders*	128 (15.7)	12 (2.8)	116 (30.8)	*p* < 0.003^a^
*Gastrointestinal disorders*	124 (15.2)	36 (8.2)	88 (23.3)	*p* < 0.003^a^
*Falls and syncope*	115 (14.1)	65 (14.9)	50 (13.3)	*p* = 0.51
Syncope	57 (9.5)	19 (4.4)	38 (10.1)	*p* < 0.003^a^
*Cardiac disorders*	107 (13.1)	87 (19.9)	20 (5.3)	*p* < 0.003^a^
Bradycardia	7 (.9)	2 (.5)	5 (1.3)	*p* = 0.18
Hypotension	17 (2.1)	3 (.7)	14 (3.7)	*p* < 0.003^a^
*Renal and urinary disorders*	87 (10.7)	45 (10.3)	42 (11.1)	*p* = 0.15
*Neurological disorders*	53 (6.5)	35 (8.0)	18 (4.8)	*p* = 0.06
Stroke	12 (1.5)	10 (2.3)	2 (0.5)	*p* = 0.04
*Muscoskeletal disorders*	32 (3.9)	27 (6.2)	5 (1.3)	*p* < 0.003^a^
*Skin and soft tissue disorders*	20 (2.5)	17 (3.9)	3 (0.8)	*p* = 0.004
*Endocrine disorders*	9 (1.1)	1 (.2)	8 (2.1)	*p* = 0.01
Hypoglycaemia	6 (0.7)	0 (0.0)	6 (1.6)	*p* = 0.01
*Electrolyte imbalance*	9 (1.1)	1 (0.2)	8 (2.1)	*p* = 0.01
*Hepatobiliary*	8 (1.0)	6 (1.4)	2 (0.5)	*p* = 0.88
*Psychiatric disorders*	4 (0.5)	2 (0.5)	2 (0.5)	*p* = 0.88
*Vascular disorders*	4 (0.5)	2 (0.5)	2 (0.5)	*p* = 0.88

^a^ Chi-squared test for categorical data with Bonferonni adjustment (*p* < 0.003). Sub-categories of primary presenting complaints at hospitalisation are the more frequent complaints within that category e.g. stroke within neurological disorders.

There was no significant difference in falls and syncope as a primary presenting issue between ADR-related hospital admissions (*n* = 50, 13.3%) and non-ADR related hospital admissions (*n* = 65, 14.9%) (p = 0.51). Further analysis found that 179 (22.0%) hospital admissions had a fall as a contributing factor (not a primary presenting issue), but again there was no significant difference between ADR-related hospital admissions (*n* = 88, 23.3%) and non-ADR related hospital admissions (*n* = 91, 20.8%) (p = 0.39).

### Risk factors associated with ADRs (per patient)

Of the 3,091 patients screened, 361 (11.7% 95% CI 10.5%, 12.8%) patients had an ADR-related admission and 47 (13.0%) of these patients experienced ≥1 ADR-related admission. Table 4 shows the main patient characteristics for the ADR and non-ADR admissions groups. In the unadjusted analysis, factors associated with an ADR-related hospital admission were having a comorbidity of cerebrovascular disease, myocardial infarction or diabetes and anticoagulant, antiplatelet, diuretic or antihypertensive medication use. Increasing age, adherence to medication and patients with a comorbidity of chronic lung disease were less likely to have an ADR-related hospital admission. In the adjusted analysis being immobile or frail, having delirium or ulcer disease and taking anticoagulant and antiplatelet medication on hospital admission were significantly associated with an ADR-related hospital admission. While older age, having state-subsidised healthcare and prescriptions (medical card), having fallen previously and having chronic lung disease were significantly associated with not having an ADR-related hospital admission.

**TABLE 4:** Unadjusted and adjusted odds ratios (OR) and 95% CI for risk factor associations with ADR-related hospital admissions and non-ADR admissions (N = 798).

Risk factors	Non-ADR admissions (*n* = 437)	ADR admissions ≥1 (*n* = 361)	Unadjusted odds ratio (95% CI)	Adjusted odds ratio (95% CI)
*Sociodemographics (N, %)*				
Age (mean, SD)	81.6 (7.7)	79.9 (7.3)	0.97 (0.95, 0.99)*	0.96 (0.94, 0.98)*
Age >85 years	153 (35.0)	103 (28.5)	0.74 (0.55, 1.00)	—
Gender- Female	233 (53.1)	184 (51.0)	0.91 (0.69, 1.20)	1.03 (0.73, 1.44)
Medical card (Yes/No)	167 (38.2)	116 (32.1)	0.77 (0.57, 1.03)	0.66 (0.47, 0.92)*
*Functional ability -Geriatric syndromes* (*N, %*)				
Immobility (Yes/No)	188 (47.4)	167 (47.9)	1.02 (0.76, 1.36)	2.33 (1.01, 5.38)*
Use of walking aids (inside) (Yes/No)	169 (42.9)	130 (37.3)	1.26 (.94, 1.69)	—
Use of walking aids (outside) (Yes/No)	192 (48.7)	163 (46.8)	1.08 (0.81, 1.44)	—
Functional impairment (Yes/No)	210 (52.9)	168 (48.1)	0.83 (0.62, 1.10)	0.71 (0.44, 1.15)
*Falls history*				
Fallen- previously (Yes/No)	203 (51.1)	169 (48.4)	0.90 (0.67, 1.20)	0.25 (0.10, 0.64)*
Fallen –in the last year (Yes/No)	132 (30.2)	111 (30.8)	1.08 (0.79, 1.46)	—
Fallen more than once (Yes/No)	61 (15.5)	50 (14.4)	1.09 (0.73, 1.64)	—
Frailty- TRS	224 (56.4)	217 (62.4)	1.28 (0.95, 1.72)	2.51 (1.39, 4.53)*
Frailty- Prisma 7	241 (60.7)	199 (57.0)	0.86 (0.64, 1.15)	—
Delirium (4AT)-Unlikely	143 (35.9)	125 (35.1)	—	—
Possible cognitive impairment	150 (37.7)	120 (33.7)	0.92 (0.65, 1.29)	
Possible delirium ± cognitive impairment	105 (26.4)	111 (31.2)	1.21 (0.84, 1.73)	
Delirium (DSM 4)	100 (25.1)	105 (29.5)	1.25 (0.90, 1.72)	1.63 (1.06, 2.50)*
Urinary incontinence (Yes/No)	4 (1.0)	26 (7.5)	—	—
Unintentional weight loss (Yes/No)	107 (27.0)	74 (21.2)	0.73 (0.52, 1.02)	0.84 (0.57, 1.24)
Nursing home resident (Yes/No)	40 (10.1)	23 (6.6)	0.63 (0.37, 1.07)	0.65 (0.34, 1.25)
Disease-related				
*Co-morbidities* vs*. none*				
Chronic lung disease	76 (17.4)	33 (19.1)	0.48 (0.31, 0.74)*	0.51 (0.28, 0.94)*
Heart failure	45 (10.3)	51 (14.1)	1.43 (0.93, 2.20)	1.17 (0.63, 2.16)
Myocardial infarction	38 (8.7)	52 (14.4)	1.77 (1.13, 2.75)*	1.34 (0.73, 2.47)
Diabetes mellitus	7 (1.6)	15 (4.2)	2.66 (1.07, 6.60)*	1.85 (0.63, 5.48)
Chronic liver disease	1 (0.2)	2 (0.6)	—	—
Cancer	3 (0.7)	4 (1.1)	—	—
Dementia	37 (8.5)	25 (6.9)	0.80 (0.47, 1.36)	1.09 (0.53, 2.25)
Cerebrovascular	38 (8.7)	48 (13.3)	1.61 (1.03, 2.52)*	1.48 (0.80, 2.73)
Chronic kidney disease	23 (5.3)	14 (3.9)	0.73 (0.37, 1.43)	0.74 (0.32, 1.71)
Connective tissue disease	18 (4.1)	12 (3.3)	0.80 (0.38, 1.68)	0.92 (0.36, 2.35)
Ulcer disease	8 (1.8)	13 (3.6)	2.00 (0.82, 4.89)	2.94 (1.04, 8.28)*
Charlson weights- 0	81 (18.6)	57 (15.8)	—	—
Charlson weights- 1 and 2	183 (42.0)	152 (42.1)	1.18 (0.79, 1.76)	1.15 (0.64, 2.07)
Charlson weights- ≥3	172 (39.5)	152 (42.1)	1.26 (0.84, 1.88)	0.97 (0.54, 1.76)
Medication-related				
No of medications (IQR)	10	10	1.02 (0.99, 1.05)	—
Polypharmacy				
Non-polypharmacy (≤4 drugs)	64 (14.7)	46 (12.7)	—	—
Polypharmacy (5–9 drugs)	211 (48.3)	164 (45.4)	1.08 (0.70, 1.66)	0.65 (0.37, 1.15)
Excessive polypharmacy (≥10 drugs)	162 (37.1)	151 (41.8)	1.30 (0.84, 2.01)	0.75 (0.38, 1.44)
*Types of medication on admission* (vs. none)				
Anticoagulants	105 (24.3)	131 (36.3)	1.80 (1.32, 2.45)*	2.00 (1.35, 2.97)*
Antiplatelets	227 (52.0)	227 (62.9)	1.57 (1.18, 2.08)*	1.64 (1.13, 2.38)*
NSAIDs	31 (7.1)	17 (4.7)	0.65 (0.35, 1.19)	0.61 (0.30, 1.24)
Diuretics	99 (22.7)	119 (33.0)	1.68 (1.23, 2.30)*	1.38 (0.95, 1.99)
Anti-hypertensives	318 (72.8)	297 (82.3)	1.74 (1.23, 2.45)*	1.41 (0.93, 2.15)
Sedatives	85 (19.5)	71 (19.7)	1.01 (0.71, 1.44)	1.18 (0.77, 1.78)
Neuroleptics	41 (9.4)	30 (8.3)	0.88 (0.53, 1.43)	0.90 (0.50, 1.64)
Antidepressants	114 (26.1)	98 (27.2)	1.06 (0.77, 1.45)	0.99 (0.66, 1.50)
Anxiolytics	52 (11.9)	35 (9.7)	0.79 (0.51, 1.25)	1.07 (0.60, 1.92)
Use of medications				
Blister pack usage (Yes/No)	155 (35.6)	134 (37.4)	0.92 (0.69, 1.23)	0.93 (0.64, 1.33)
Self-reported adherence (Yes)	436 (100)	336 (93.3)	0.77 (0.67, 0.89)	-

*p-value < 0.05. Use of walking aids, fallen in previous year or more than once, delirium (4AT), number of medications and self-reported adherence were omitted from the multivariable analysis because of collinearity. Urinary incontinence, chronic liver disease and cancer were omitted from the analysis as n < 5 in ADR, or non-ADR, group. 737 patients were included in the multivariable analysis (data were missing at random for 61 patients).

## Discussion

### Summary of main findings

This study found that 10.0% of hospital admissions in older patients (≥65 years) in a large tertiary referral hospital in Ireland were ADR-related, with 58.1% of these admissions caused by an ADR and ADRs contributing to the remaining admissions. Furthermore, approximately 71% of ADR-related admissions were deemed definitely or possibly preventable/avoidable. Antithrombotic agents, mainly aspirin and warfarin, were the drugs most frequently associated with ADR-related hospital admissions. A number of cardiovascular system drugs and psychoanaleptics were also associated with ADR-related hospital admissions. There was a significantly higher proportion of bleeding disorders, syncope, gastrointestinal disorders and hypotension in ADR-related hospital admissions, compared to non-ADR hospital admissions. Immobility, frailty, delirium, having a comorbidity of ulcer disease and taking anticoagulant and antiplatelet medication on hospital admission were independently associated with an ADR-related hospital admission.

The prevalence of ADR-related hospital admissions, as observed in our study, is higher than that of a previous Irish study (Ahern et al., 2014) but is consistent with other studies, which have reported that approximately 10% of admissions are ADR-related (Kongkaew et al., 2008; Alhawassi et al., 2014). Almost three-quarters of the ADR-related hospital admissions were deemed potentially avoidable which suggests a considerable opportunity to reduce healthcare burden and costs due to ADRs (Pirmohamed et al., 2004). There was good agreement on the assessment of ADR causality among the review panel using three algorithms. Higher inter-rater agreement has been reported when using algorithms *versus* clinical judgement, but no one measure is universally accepted (Agbabiaka et al., 2008).

Medicines which have been particularly implicated in ADR-related hospital admissions include antiplatelets, anticoagulants, NSAIDs, cytotoxics, immunosuppressants, diuretics, antidiabetics and antibiotics (Howard et al., 2007; Coleman and Pontefract, 2016). These medications have a high innate toxicity, particularly in older populations (Howard et al., 2007). A previous study of adverse drug events (ADE) in primary care in Ireland found that 86% of patients prescribed aspirin and warfarin reported bruising, bleeding, and indigestion (Cahir et al., 2019). In the current study, of ADR-related hospital admissions these drugs were associated with gastrointestinal and vascular haemorrhage. In a US study of older adults, gastrointestinal haemorrhage was one of the most frequently reported ADRs (Budnitz et al., 2011), while in the United Kingdom, 20 out of 28 deaths in ADR-related hospital admissions were due to gastrointestinal or intracranial bleeding (Pirmohamed et al., 2004). Antithrombotic agents such as warfarin need to be carefully monitored and titrated according to the international normalised ratio (INR) in older people to reduce ADR-related hospital admissions (Bloomfield et al., 2011). Consistent with previous studies, cardiovascular and psychotropic drugs contributed to a large number of ADR-related hospital admissions, most commonly renal impairment and electrolyte disturbances (Pedrós et al., 2014; Lucenteforte et al., 2017). In Italy, 39.5% of suspected ADE-related hospitalisations in the older population were related to cardiovascular medications, including beta-blockers, diuretics and renin-angiotensin system inhibitors (Crescioli et al., 2021). To avoid hypotensive episodes, guidelines recommend adopting an individualised holistic approach in deciding on blood pressure targets, particularly in those aged ≥80 years and with significant postural hypotension (Benetos et al., 2019; Kulkarni et al., 2020).

A number of risk factors have previously being identified as associated with ADRs in older populations, including female sex, advanced age, increased disease burden, number of medications and polypharmacy (Alhawassi et al., 2014). In the current study these factors were not significantly associated with ADR-related hospital admissions. Differences in this study in identified risk factors may be due to the cohort comprising of older, frail patients with multiple comorbidities who were prescribed on average ten or more medications. Identifying independent risk factors for ADR-related hospital admissions is particularly challenging as both risk factors and ADRs can present as non-specific symptoms and syndromes that are highly prevalent in older people (e.g. delirium, falls, ulcer disease) (Davies and O'Mahony, 2015). In a US study of long-term care residents, delirium was identified as one of the most common indications of a potential ADE and a trigger for medication rationalisation (Wierenga et al., 2012). On the other hand, polypharmacy has been identified as a risk factor for delirium, but it is unclear which medications or medication combinations are implicated. (Clegg and Young, 2011).

### Strengths and limitations

This study is one of the first large scale studies on ADR-related hospital admissions in Ireland. The large sample size enabled the study to establish detailed information on the characterisation of ADRs and related drugs, patient morbidity and functional status from a number of sources. A gold-standard medication reconciliation list was completed, where the patients’ medication list was verified by a pharmacist against two alternative sources (Almanasreh et al., 2016). Nearly all consecutive hospitalisations in older people due to acute illnesses were included, reducing selection bias. The causality, preventability and severity of each ADR and the contribution of the ADR to hospitalisation were independently investigated by two investigators based on standard criteria.

However, there are a number of limitations. The study was conducted in a single large hospital and the results may, therefore, not be generalizable to other settings. While, the determination of ADR prevalence included a multifaceted review of each suspected ADR including clinical judgement and chart review, and the application of a number of validated algorithms, there is a potential risk of misclassification, particularly as the study population had several comorbidities and disabilities and were prescribed a large number of medications.

### Implications

The prevalence of ADR-related hospital admissions is high in older populations and many of these ADRs are deemed preventable. ADRs should be considered as a potential diagnosis in older complex patients, especially where symptom presentation is non-specific (Davies and O'Mahony, 2015). Not recognising an ADR in clinical practice may lead to a prescribing cascade whereby a new drug is prescribed to treat the adverse effects of an existing drug, potentially leading to further adverse health outcomes for the patient (Lavan and Gallagher, 2016). There is a lack of reliable and valid ADR detection and prediction tools developed for use in community settings. Current ADR causality tools are difficult to apply in everyday practice and inter-rater reliability amongst the tools is not robust (Agbabiaka et al., 2008). Predictive factors for ADR-related hospital admissions are still poorly understood. While some ADR risk prediction tools have been developed (e.g. ADRROP, GerontoNet), their predictive validity is low, and they are not universally accepted or used routinely in clinical practice (Lavan and Gallagher, 2016; Stevenson et al., 2014; O'Mahony et al., 2018). The focus to date has mainly being on investigating patient factors and further research needs to be completed to tease out the complex relationship between particular high-risk drug classes, multimorbidity, frailty and ADR-related hospital admissions (Jennings et al., 2019). The tools also need to be practical and efficient to use in clinical practice and the focus may need to be narrowed to specific high-risk drugs or drug class combinations.

Reliable, valid and user-friendly methods to detect and predict ADRs in community settings are necessary in order to develop interventions to reduce ADR-related hospital admissions. Improved therapeutic monitoring and pharmacotherapeutic adjustments, appropriate deprescribing and medication reviews have all being identified as interventions to minimise ADR-related admissions in older populations (Angamo et al., 2016; Gray et al., 2018). Empowering older patients through health education and literacy may also reduce the burden of ADR-related hospital admissions (Cahir et al., 2019). Literature reviews have highlighted the importance of patient involvement and shared decision-making in medication reviews and deprescribing but acknowledge that their implementation in clinical practice is complex and challenging (Reeve et al., 2014; Scott et al., 2015).

In conclusion, ADR-related hospital admissions in older people are a common clinical problem resulting in significant morbidity, healthcare consumption and costs (Wu et al., 2012). They are largely preventable through improved pharmacological management and education. Future research needs to focus on developing community-based tools and skills to enable healthcare providers and older patients detect and differentiate adverse effects of medication from symptoms of chronic disease or frailty and to identify those most at risk of medication-related harm. This may ultimately reduce ADR-related hospital admissions in the ever increasing population of older multi-morbid adults.

## Data Availability

The dataset presented in this article is not publicly available for use. Requests to access the dataset should be directed to caitrionacahir@rcsi.ie.

## References

[B1] Agbabiaka T. B. Savović J. Ernst E. (2008). Methods for causality assessment of adverse drug reactions: A systematic review. Drug Saf. 31 (1), 21–37. 10.2165/00002018-200831010-00003 18095744

[B2] Ahern F. Sahm L. J. Lynch D. McCarthy S. (2014). Determining the frequency and preventability of adverse drug reaction-related admissions to an Irish university hospital: A cross-sectional study. Emerg. Med. J. 31 (1), 24–29. 10.1136/emermed-2012-201945 23389832

[B3] Alhawassi T. M. Krass I. Bajorek B. V. Pont L. G. (2014). A systematic review of the prevalence and risk factors for adverse drug reactions in the elderly in the acute care setting. Clin. Interventions Aging 9, 2079–2086. 10.2147/CIA.S71178 PMC4257024 25489239

[B4] Almanasreh E. Moles R. Chen T. F. (2016). The medication reconciliation process and classification of discrepancies: A systematic review. Br. J. Clin. Pharmacol. 82 (3), 645–658. 10.1111/bcp.13017 27198753 PMC5338112

[B5] Altman D. G. (1991). Practical statistics for medical research. London: Chapman & Hall.

[B6] American Psychiatric Association (1994). Diagnostic and statistical manual of mental disorders. 4th edition. Washington, DC: American Psychiatric Association.

[B7] Angamo M. T. Chalmers L. Curtain C. M. Bereznicki L. R. (2016). Adverse-drug-reaction-related hospitalisations in developed and developing countries: A review of prevalence and contributing factors. Drug Saf. 39 (9), 847–857. 10.1007/s40264-016-0444-7 27449638

[B8] Benetos A. Petrovic M. Strandberg T. (2019). Hypertension management in older and frail older patients. Circulation Res. 124 (7), 1045–1060. 10.1161/CIRCRESAHA.118.313236 30920928

[B9] Bloomfield H. E. Krause A. Greer N. Taylor B. C. MacDonald R. Rutks I. (2011). Meta-analysis: Effect of patient self-testing and self-management of long-term anticoagulation on major clinical outcomes. Ann. Intern Med. 154 (7), 472–482. 10.7326/0003-4819-154-7-201104050-00005 21464349

[B10] Budnitz D. S. Lovegrove M. C. Shehab N. Richards C. L. (2011). Emergency hospitalizations for adverse drug events in older Americans. N. Engl. J. Med. 365 (21), 2002–2012. 10.1056/NEJMsa1103053 22111719

[B11] Cahir C. Curran C. Byrne C. Walsh C. Hickey A. Williams D. J. (2017). Adverse drug reactions in an ageing PopulaTion (ADAPT) study protocol: A cross-sectional and prospective cohort study of hospital admissions related to adverse drug reactions in older patients. BMJ Open 7 (6), e017322. 10.1136/bmjopen-2017-017322 PMC5726049 28600381

[B12] Cahir C. Wallace E. Cummins A. Teljeur C. Byrne C. Bennett K. (2019). Identifying adverse drug events in older community-dwelling patients. Ann. Fam. Med. 17 (2), 133–140. 10.1370/afm.2359 30858256 PMC6411408

[B13] Charlson M. Pompei P. Ales K. MacKenzie C. (1987). A new method of classifying prognostic comorbidity in longitudinal studies: Development and validation. J. Chronic Dis. 40, 373–383. 10.1016/0021-9681(87)90171-8 3558716

[B14] Clegg A. Young J. B. (2011). Which medications to avoid in people at risk of delirium: A systematic review. Age Ageing 40 (1), 23–29. 10.1093/ageing/afq140 21068014

[B15] Coleman J. J. Pontefract S. K. (2016). Adverse drug reactions. Clin. Med. Lond. Engl. 16 (5), 481–485. 10.7861/clinmedicine.16-5-481 PMC6297296 27697815

[B16] Crescioli G. Bettiol A. Bonaiuti R. Tuccori M. Rossi M. Capuano A. (2021). Risk of hospitalization associated with cardiovascular medications in the elderly Italian population: A nationwide multicenter study in emergency departments. Front. Pharmacol. 11, 61110. 10.3389/fphar.2020.61110 PMC7941274 33708120

[B17] Davies E. A. O'Mahony M. S. (2015). Adverse drug reactions in special populations - the elderly. Br. J. Clin. Pharmacol. 80 (4), 796–807. 10.1111/bcp.12596 25619317 PMC4594722

[B18] De J. Wand A. P. Smerdely P. I. Hunt G. E. (2016). Validating the 4A's test in screening for delirium in a culturally diverse geriatric inpatient population. Int. J. geriatric psychiatry 32, 1322–1329. 10.1002/gps.4615 27766672

[B19] Dwyer M. Peklar J. McCallion P. McCarron M. Henman M. C. (2016). Factors associated with polypharmacy and excessive polypharmacy in older people with intellectual disability differ from the general population: A cross-sectional observational nationwide study. BMJ Open 6 (4), e010505. 10.1136/bmjopen-2015-010505 PMC4823458 27044582

[B20] Edwards I. R. Aronson J. K. (2000). Adverse drug reactions: Definitions, diagnosis, and management. Lancet 356 (9237), 1255–1259. 10.1016/S0140-6736(00)02799-9 11072960

[B21] Fan J. Worster A. Fernandes C. M. (2006). Predictive validity of the triage risk screening tool for elderly patients in a Canadian emergency department. Am. J. Emerg. Med. 24 (5), 540–544. 10.1016/j.ajem.2006.01.015 16938591

[B22] Franceschi M. Scarcelli C. Niro V. Seripa D. Pazienza A. M. Pepe G. (2008). Prevalence, clinical features and avoidability of adverse drug reactions as cause of admission to a geriatric unit: A prospective study of 1756 patients. Drug Saf. 31 (6), 545–556. 10.2165/00002018-200831060-00009 18484788

[B23] Gallagher R. M. Kirkham J. J. Mason J. R. Bird K. A. Williamson P. R. Nunn A. J. (2011). Development and inter-rater reliability of the Liverpool adverse drug reaction causality assessment tool. PLOS ONE 6 (12), e28096. 10.1371/journal.pone.0028096 22194808 PMC3237416

[B24] Gray S. L. Hart L. A. Perera S. Semla T. P. Schmader K. E. Hanlon J. T. (2018). Meta-analysis of interventions to reduce adverse drug reactions in older adults. J. Am. Geriatr. Soc. 66 (2), 282–288. 10.1111/jgs.15195 29265170 PMC5809283

[B25] Hallas J. Harvald B. Gram L. F. Grodum E. Brosen K. Haghfelt T. (1990). Drug related hospital admissions: The role of definitions and intensity of data collection, and the possibility of prevention. J. Intern Med. 228 (2), 83–90. 10.1111/j.1365-2796.1990.tb00199.x 2394974

[B26] Hartwig S. Siegel J. Schneider P. (1992). Preventability and severity assessment in reporting adverse drug reactions. Am. J. Health-System Pharm. 49 (9), 2229–2232. 10.1093/ajhp/49.9.2229 1524068

[B27] Hebert R. Raiche M. Dubois M. F. Gueye N. R. Dubuc N. Tousignant M. (2010). Impact of PRISMA, a coordination-type integrated service delivery system for frail older people in quebec (Canada): A quasi-experimental study. journals gerontology Ser. B, Psychol. Sci. Soc. Sci. 65b (1), 107–118. 10.1093/geronb/gbp027 19414866

[B28] Hopf Y. Watson M. Williams D. (2008). Adverse-drug-reaction related admissions to a hospital in Scotland. Pharm. World & Sci. 30 (6), 854–862. 10.1007/s11096-008-9240-5 18654836

[B29] Howard R. L. Avery A. J. Slavenburg S. Royal S. Pipe G. Lucassen P. (2007). Which drugs cause preventable admissions to hospital? A systematic review. Br. J. Clin. Pharmacol. 63 (2), 136–147. 10.1111/j.1365-2125.2006.02698.x 16803468 PMC2000562

[B30] Jennings E. Gallagher P. O’Mahony D. (2019). Detection and prevention of adverse drug reactions in multi-morbid older patients. Age Ageing 48 (1), 10–13. 10.1093/ageing/afy157 30299453

[B31] Kongkaew C. Noyce P. R. Ashcroft D. M. (2008). Hospital admissions associated with adverse drug reactions: A systematic review of prospective observational studies. Ann. Pharmacother. 42 (7), 1017–1025. 10.1345/aph.1L037 18594048

[B32] Kulkarni A. M. A. Yang E. Parapid B. (2020). Older adults and hypertension: Beyond the 2017 guideline for prevention, detection, evaluation, and management of high blood pressure in adults. US: American College of Cardiology. Expert Analysis.

[B33] Lavan A. H. Gallagher P. (2016). Predicting risk of adverse drug reactions in older adults. Ther. Adv. Drug Saf. 7 (1), 11–22. 10.1177/2042098615615472 26834959 PMC4716390

[B34] Lehnert T. Heider D. Leicht H. Heinrich S. Corrieri S. Luppa M. (2011). Review: Health care utilization and costs of elderly persons with multiple chronic conditions. Med. Care Res. Rev. 68 (4), 387–420. 10.1177/1077558711399580 21813576

[B35] Lucenteforte E. Lombardi N. Vetrano D. L. La Carpia D. Mitrova Z. Kirchmayer U. (2017). Inappropriate pharmacological treatment in older adults affected by cardiovascular disease and other chronic comorbidities: A systematic literature review to identify potentially inappropriate prescription indicators. Clin. Interv. Aging 12, 1761–1778. 10.2147/CIA.S137403 29089750 PMC5656349

[B36] Naranjo C. A. Busto U. Sellers E. M. Sandor P. Ruiz I. Roberts E. A. (1981). A method for estimating the probability of adverse drug reactions. Clin. Pharmacol. Ther. 30 (2), 239–245. 10.1038/clpt.1981.154 7249508

[B37] O'Mahony D. O'Connor M. N. Eustace J. Byrne S. Petrovic M. Gallagher P. (2018). The adverse drug reaction risk in older persons (ADRROP) prediction scale: Derivation and prospective validation of an ADR risk assessment tool in older multi-morbid patients. Eur. Geriatr. Med. 9 (2), 191–199. 10.1007/s41999-018-0030-x 34654257

[B38] Onder G. Petrovic M. Tangiisuran B. Meinardi M. C. Markito-Notenboom W. P. Somers A. (2010). Development and validation of a score to assess risk of adverse drug reactions among in-hospital patients 65 years or older: The GerontoNet ADR risk score. Archives Intern. Med. 170 (13), 1142–1148. 10.1001/archinternmed.2010.153 20625022

[B39] Osanlou R. Walker L. Hughes D. A. Burnside G. Pirmohamed M. (2022). Adverse drug reactions, multimorbidity and polypharmacy: A prospective analysis of 1 month of medical admissions. BMJ Open 12 (7), e055551. 10.1136/bmjopen-2021-055551 PMC9255409 35788071

[B40] Oscanoa T. J. Lizaraso F. Carvajal A. (2017). Hospital admissions due to adverse drug reactions in the elderly. A meta-analysis. Eur. J. Clin. Pharmacol. 73 (6), 759–770. 10.1007/s00228-017-2225-3 28251277

[B41] Parameswaran Nair N. Chalmers L. Bereznicki B. J. Curtain C. M. Bereznicki L. R. (2017). Repeat adverse drug reaction-related hospital admissions in elderly Australians: A retrospective study at the royal hobart hospital. Drugs Aging 34 (10), 777–783. 10.1007/s40266-017-0490-6 28952130

[B42] Pedrós C. Quintana B. Rebolledo M. Porta N. Vallano A. Arnau J. M. (2014). Prevalence, risk factors and main features of adverse drug reactions leading to hospital admission. Eur. J. Clin. Pharmacol. 70 (3), 361–367. 10.1007/s00228-013-1630-5 24362489

[B43] Pirmohamed M. James S. Meakin S. Green C. Scott A. K. Walley T. J. (2004). Adverse drug reactions as cause of admission to hospital: Prospective analysis of 18 820 patients. BMJ 329 (7456), 15–19. 10.1136/bmj.329.7456.15 15231615 PMC443443

[B44] Reeve E. Shakib S. Hendrix I. Roberts M. S. Wiese M. D. (2014). Review of deprescribing processes and development of an evidence-based, patient-centred deprescribing process. Br. J. Clin. Pharmacol. 78 (4), 738–747. 10.1111/bcp.12386 24661192 PMC4239968

[B45] Sari A. B-A. Sheldon T. A. Cracknell A. Turnbull A. (2007). Sensitivity of routine system for reporting patient safety incidents in an NHS hospital: Retrospective patient case note review. BMJ 334 (7584), 79. 10.1136/bmj.39031.507153.AE 17175566 PMC1767248

[B46] Scott I. A. Hilmer S. N. Reeve E. Potter K. Le Couteur D. Rigby D. (2015). Reducing inappropriate polypharmacy: The process of deprescribing. JAMA Intern. Med. 175 (5), 827–834. 10.1001/jamainternmed.2015.0324 25798731

[B47] Sinnott S-J. Bennett K. Cahir C. (2017). Pharmacoepidemiology resources in Ireland—An introduction to pharmacy claims data. Eur. J. Clin. Pharmacol. 73, 1449–1455. 10.1007/s00228-017-2310-7 28819675 PMC5662670

[B48] Stevenson J. Williams J. L. Burnham T. G. Prevost A. T. Schiff R. Erskine S. D. (2014). Predicting adverse drug reactions in older adults; a systematic review of the risk prediction models. Clin. Interventions Aging 9, 1581–1593. 10.2147/CIA.S65475 PMC4178502 25278750

[B49] Waller P. Shaw M. Davidson H. Shakir S. Ebrahim S. (2004). Hospital admissions for 'drug-induced' disorders in england: A study using the hospital episodes statistics (HES) database. Br. J. Clin. Pharmacol. 59 (2), 213–219. 10.1111/j.1365-2125.2004.02236.x PMC1884760 15676044

[B50] WHO (2005). The WHO adverse reaction terminology – WHO-art. Sweden: The Uppsala Monitoring Center.

[B51] Wierenga P. C. Buurman B. M. Parlevliet J. L. van Munster B. C. Smorenburg S. M. Inouye S. K. (2012). Association between acute geriatric syndromes and medication-related hospital admissions. Drugs Aging 29 (8), 691–699. 10.2165/11632510-000000000-00000 22812539 PMC3622589

[B52] Williams D. J. Olsen S. Crichton W. Witte K. Flin R. Ingram J. (2008). Detection of adverse events in a Scottish hospital using a consensus-based methodology. Scott. Med. J. 53 (4), 26–30. 10.1258/RSMSMJ.53.4.26 19051661

[B53] Wu C. Bell C. M. Wodchis W. P. (2012). Incidence and economic burden of adverse drug reactions among elderly patients in ontario emergency departments: A retrospective study. Drug Saf. 35 (9), 769–781. 10.1007/BF03261973 22823502 PMC3714138

