# Supplementary material for: Adverse drug reactions in an ageing PopulaTion (ADAPT) study: Prevalence and risk factors associated with adverse drug reaction-related hospital admissions in older patients
Source: Front Pharmacol. 2023 Jan 13;13:1029067. doi: 10.3389/fphar.2022.1029067 (PMC9880441; doi:10.3389/fphar.2022.1029067)
Supplement: Supplementary file 1 [file Table1.pdf]

Supplementary Table 1

S1: ADR-related hospital admissions per main diagnostic category (N=424)

**Diagnostic category**	**N (%)**	**Condition**	**N (%)**
**Renal and urinary Disorders**	85 (20.1)	Renal failure and renal impairment	85 (20.1)
**Vascular Disorders**	97 (22.9)	*Haemorrhages:*	
		Epistaxis	10 (2.4)
		Haematuria	21 (5.0)
		Haematoma	10 (2.4)
		Vascular hypotensive disorders	50 (11.8)
**Metabolism and nutrition disorders**	80 (18.9)	*Glucose metabolism disorder*	
		Hypoglycaemic conditions	11 (2.6)
		Electrolyte and fluid balance conditions	
		Hyponatraemia	57 (13.4)
		Hypokalaemia	6 (1.4)
**Cardiac Disorders**	14 (3.3)	Bradycardia	11 (2.6)
**Investigations**	11 (2.6)	Elevated INR	10 (2.4)
**Blood and lymphatic system disorders**	9 (2.1)	Neutropenia	9 (2.1)
**Gastrointestinal**	84 (19.8)	*Gastrointestinal haemorrhage*	
		Gastric and oesophageal haemorrhage	36 (8.5)
		Intestinal haemorrhage	22 (5.2)
		Non site specific haemorrhage	14 (3.3)
		Gastrointestinal motility and defecation conditions	
		Diarrhoea unspecified	5 (1.2)
**Immune System Disorders**	10 (2.4)	Secondary immunodeficiency	7 (1.7)
**Nervous system Disorders**	24 (5.7)	*Central nervous system haemorrhages and cerebrovascular accidents*	
		Cerebral haemorrhage	8 (1.9)
		Subdural haemorrhages	5 (1.2)
		Neurological disorders	
		Somnolence	5 (1.2)

The diagnostic categories and conditions are reported using Medical Dictionary for Regulatory Activities (MedDRA) terminology
